# Midline suboccipital craniotomy and direct stimulation for a dorsally exophytic brainstem tumor

**DOI:** 10.3171/2019.10.FocusVid.19456

**Published:** 2019-10-01

**Authors:** David S. Hersh, Katherine N. Sanford, Kenneth Moore, Frederick A. Boop

**Affiliations:** 1 Department of Neurosurgery, University of Tennessee Health Science Center;; 2 Le Bonheur Children’s Hospital; and; 3 Semmes Murphey Clinic, Memphis, Tennessee

**Keywords:** brainstem, exophytic, stimulation, neuromonitoring, video

## Abstract

Dorsally exophytic brainstem tumors arise from within the brainstem itself. As the tumor grows, it pulls eloquent tissue with it, resulting in a shape that is analogous to the sides of a volcano. Rather than a resection that is flush with the brainstem being performed, this functional tissue on the lateral edges of the tumor must be identified and preserved in order to avoid postoperative deficits. The authors describe a midline, suboccipital approach with the use of intraoperative direct stimulation to identify and preserve functional tissue innervating the palate during the resection of a dorsally exophytic medullary tumor.

The video can be found here: https://youtu.be/qbk2DvInO8o.

## Transcript

In this video, we describe a midline, suboccipital approach with the use of intraoperative direct stimulation to identify and preserve functional tissue during the resection of a dorsally exophytic medullary tumor.

### 0:37 History

The patient was a 5-year-old female who was initially referred for scoliosis. Imaging was performed during the course of her workup, revealing a dorsally exophytic medullary tumor extending into the cisterna magna.

### 0:53 Preop imaging

T1- and T2-weighted MRI sequences demonstrated a 2.1 × 2.6 × 2.8–cm enhancing lesion arising from the dorsal aspect of the medulla. The lesion was exophytic into the cisterna magna, and the enhancing portion of the lesion demonstrated heterogeneous diffusion characteristics with predominantly facilitated diffusion. Diffusion tensor fiber tracking demonstrated no clear evidence of direct involvement of the pyramidal tracts.

### 1:29 Positioning and opening

The patient was placed in the MRI-compatible carbon graphite pediatric pin head holder and turned to the prone position with the chin flexed. Needle electrodes were placed for intraoperative neurophysiological monitoring, including brainstem evoked potentials, somatosensory evoked potentials, and lower cranial nerve monitoring. A midline suboccipital incision was used to dissect the soft tissues from the inion to the top of C2. A suboccipital craniotomy and a C1 laminectomy were performed and the dura was opened in a Y-shaped fashion.

### 2:08 Biopsies

Once dural tackup stitches were placed, the microscope was brought into the field and the arachnoid overlying the cisterna magna was resected. The capsule of the tumor was sharply incised. Biopsies were taken from the apex of the tumor. Frozen specimens were prepared and revealed pathological findings consistent with a pilocytic astrocytoma.

### 2:39 Dissection

Using microsurgical dissection techniques, we began dissecting around the base of the tumor. Along the edges of the tumor, the white matter had been expanded by the dorsally exophytic growth of the tumor. We used direct stimulation, beginning at 1 mA, and identified responses from the muscles of the palate. Using this localization data, we continued to dissect around the tumor using a combination of bipolar coagulation and sharp dissection, taking additional care in areas where functional tissue was identified. As we dissected around the tumor, Gelfoam and cottonoids were placed circumferentially.

### 3:54 Debulking and further dissection

We then began debulking the tumor with the ultrasonic aspirator. Once the tumor had been sufficiently debulked, we were able to continue dissecting around the far side of the tumor. We stopped periodically to confirm where we were using intraoperative neuronavigation and direct stimulation. We came to a point where the obvious yellow, gelatinous tumor began to blend with the white matter of the medulla. At this point, the resection was stopped. We were able to dissect around the tumor rostrally to free up the foramen of Magendie, in which Gelfoam was placed in order to prevent rundown of blood products. Tumor was then further debulked at that level using the ultrasonic aspirator.

Finally, the cavity was inspected and meticulous hemostasis was secured. All Gelfoam and cottonoids were removed. The wound was irrigated until clear. The intraoperative monitoring electrodes were removed, the patient was covered with a sterile drape, and an intraoperative MRI was performed.

### 6:00 Intraoperative imaging

Intraoperative MRI revealed resection of the previously seen dorsally exophytic mass. The exophytic portion was resected, with restoration of patency of the cisterna magna and improved patency of the foramen of Magendie. Along the inferior aspect of the tumor, a small residual area of enhancement was visualized. This imaging was consistent with a near-total resection.

### 6:29 Closing

Meticulous hemostasis was obtained and the wound was irrigated. The dura was closed with running sutures incorporating a bovine pericardial patch graft. A watertight closure was confirmed, and Gelfoam soaked in bacitracin was placed in the epidural space. The bone flap was resecured with titanium plates and screws. The wound was then closed in multiple layers.

### 6:59 Postoperative exam

The patient experienced some postoperative neck stiffness, which resolved within 2 weeks. Her neurological exam remained intact, including her gag reflex, swallowing function, gait, and sleep pattern. Here, she can be seen during a visit to Graceland 4 days postoperatively.

### 7:23 Follow-up imaging

Follow-up imaging confirmed a near-total resection, and the histology demonstrated a pilocytic astrocytoma, for which the patient is currently undergoing close postoperative surveillance.

### 7:37 Growth of a dorsally exophytic brainstem tumor

Epstein and Farmer (1993) observed that fiber tracts and pial borders direct the growth of low-grade brainstem lesions. A benign lesion originating in the brainstem initially grows within the substance of the medulla, causing swelling. However, this region is bordered anteriorly and laterally by pia, superiorly by a pontomedullary barrier consisting of decussating transverse fibers—the pontocerebellar tracts, and inferiorly by a cervicomedullary barrier consisting of anatomical structures that include the pyramidal decussations, internal arcuate fibers, and medial lemniscus. It therefore begins to grow in the direction of least resistance along its dorsal aspect, which is lined by a softer barrier, the ependyma of the fourth ventricle. The tumor ultimately becomes dorsally exophytic (Epstein and Farmer, 1993). This model of tumor growth highlights the concept that dorsally exophytic brainstem tumors arise from within the brainstem itself, and that as the tumor grows, it pulls eloquent tissue with it.

### 8:54 Volcano analogy

This results in a shape that is analogous to the sides of a volcano. Here, we have demonstrated that rather than performing a resection that is flush with the brainstem, this functional tissue on the lateral edges of the tumor must be identified and preserved in order to avoid postoperative deficits.
